# Two decades of liver resection with a multidisciplinary approach in a single institution: What has changed? Analysis of 1409 cases

**DOI:** 10.1016/j.clinsp.2022.100088

**Published:** 2022-07-25

**Authors:** Paulo Herman, Gilton Marques Fonseca, Fabricio Ferreira Coelho, Jaime Arthur Pirola Kruger, Fabio Ferrari Makdissi, Vagner Birk Jeismann, Flair José Carrilho, Luiz Augusto Carneiro D'Albuquerque, Sergio Carlos Nahas

**Affiliations:** Serviço de Cirurgia do Fígado, Departamento de Gastroenterologia, Hospital das Clínicas, Faculdade de Medicina, Universidade de São Paulo (HCFMUSP), São Paulo, SP, Brazil

**Keywords:** Hepatectomy, Liver neoplasms, Hepatocellular carcinoma, Colorectal liver metastases, Morbidity

## Abstract

**Objectives:**

To evaluate results of patients undergoing liver resection in a single center over the past two decades with a particular look at Colorectal Liver Metastasis (CRLM) and Hepatocellular Carcinoma (HCC).

**Method:**

Patients were divided into two eras, from 2000 to 2010 (Era 1) and 2011 to 2020 (Era 2). The most frequent diagnosis was CRLM and HCC, with 738 (52.4%) and 227 (16.1%) cases respectively. An evaluation of all liver resection cases and a subgroup analysis of both CRLM and HCC were performed. Preoperative and per operative variables and long-term outcomes were evaluated.

**Results:**

1409 liver resections were performed. In Era 2 the authors observed higher BMI, more: minimally invasive surgeries, Pringle maneuvers, and minor liver resections; and less transfusion, less ICU necessity, and shorter length of hospital stay. Severe complications were observed in 14.7% of patients, and 90-day mortality was 4.2%. Morbidity and mortality between eras were not different. From 738 CRLM resections, in Era 2 there were significantly more patients submitted to neoadjuvant chemotherapy, bilateral metastases, and smaller sizes with significantly less transfusion, the necessity of ICU, and shorter length of hospital stay. More pedicle clamping, minimally invasive surgeries, and minor resections were also observed. From 227 HCC resections, in Era 2 significantly more minimally invasive surgeries, fewer transfusions, less necessity of ICU, and shorter length of hospital stay were observed. OS was not different between eras for CRLM and HCC.

**Conclusions:**

Surgical resection in a multidisciplinary environment remains the cornerstone for the curative treatment of primary and metastatic liver tumors.

## Highlights

• Analysis of changes in hepatectomies during the last 2 decades.
• The surgical treatment of liver tumors needs a multidisciplinary approach.

## Introduction

Historically, liver resection was considered a complex procedure followed by high mortality rates. In the 1970s, mortality rates up to 10%‒20% were observed.^1^^,^^2^ However, with the increase in experience and the formation of centers dedicated to liver surgery, a significant improvement in the safety of hepatic resection was observed. Recent series from high-volume specialized centers have reported mortality rates of < 3‒5%.^3^, ^4^, ^5^ As a result, hepatic resection has evolved into the treatment of choice for selected patients with benign and malignant hepatobiliary disease.^2^^,^^4^^,^^6^

The main indication for liver resection in Western countries is Colorectal Cancer Liver Metastasis (CRLM). Hepatic resection is the only potentially curative therapy for selected patients with CRLM. Large single centers, as well as multi-institutional experiences, have shown 5-year overall survival rates ranging from 35% to 57% following liver resection.^5^^,^^7^, ^8^, ^9^ Better image tools for liver and extra-hepatic staging, novel surgical strategies such as parenchyma preserving resection, selective portal vein embolization, liver venous deprivation, 2-stage hepatectomy, ALPPS, and the possibility of extrahepatic tumor eradication, have allowed patients with multiple nodules and even large tumor burden to undergo complete resection.^5^^,^^10^, ^11^, ^12^, ^13^, ^14^ In the last years, systemic chemotherapy new regimens could provide significant response rates in the majority of patients, including those with an initially unresectable disease which, after excellent response, became resectable (conversion therapy).^15^^,^^16^ In addition, response to chemotherapy treatment is a surrogate factor of better tumor biology, used for selecting patients for resection.^17^^,^^18^ All these strategies, associated with a better selection of patients, have led to an expansion of the indications for CRLM resection.

In Eastern countries, the main indication for liver resection is Hepatocellular Carcinoma (HCC), the most common primary liver cancer. The available curative therapies for HCC are a liver transplant, resection, and ablation. Liver transplantation is suitable for patients with impaired liver function and portal hypertension within selective criteria (Milan criteria), ablation is reserved for nodules < 2 cm in diameter, and resection is indicated for patients with preserved liver function.^19^^,^^20^ Resection proved to be a safe procedure in the last years with good long-term results (50%‒70%, 5-year overall survival) and mortality rates in specialized centers lower than 7%.^20^, ^21^, ^22^ Compared to liver transplantation, resection is immediately available, not limited to restrictive indication criteria, present lower costs, and offers a surgical specimen for evaluation. Moreover, it does not preclude rescue transplantation.^23^

The practice of liver surgery continues to evolve, especially in a multidisciplinary context. The present study evaluates the features, trends, and perioperative and long-term results of consecutive patients undergoing liver resection in a single center over the past two decades with a particular look at CRLM and HCC.

## Methods

A retrospective study of all consecutive patients who underwent liver resection at our institution between 2000 and 2020 was performed. Data was collected using REDCap electronic database.^24^ This study was approved by the institutional Ethics Committee.

Exclusion criteria were patients subjected to first-step liver resection for staged hepatectomy that did not reach the second step; liver cysts defenestration.

In order to evaluate what has changed over the past 20 years, patients were divided according to two different eras, from 2000 to 2010 (Era 1), and from 2011 to 2020 (Era 2). The most frequent diagnosis in all series was Colorectal Liver Metastasis (CRLM) and Hepatocellular Carcinoma (HCC), with 738 (52.4%) and 227 (16.1%) cases respectively. An evaluation of all liver resection cases and a subgroup analysis of both diagnoses, CRLM and HCC was then performed.

All cases were previously discussed at a multidisciplinary meeting where surgery was indicated. For CRLM the inclusion criteria were complete resection of all hepatic lesions, liver remnant > 25% in healthy livers and > 30% after long-term chemotherapy, and limited resectable extra-hepatic disease. All patients with CRLM were submitted to perioperative chemotherapy. For HCC the inclusion criteria were uni or oligonodular disease (up to 3 nodules), absence of extrahepatic disease, Child-Pugh A (or B when minor peripheral resection was required), and Model of End-Stage Liver Disease (MELD) ≤ 10, without clinically significant portal hypertension (small caliber esophageal varices and platelets > 100.000 mL), and future liver remnant ≥ 40%.

The authors evaluated preoperative and perioperative variables and long-term outcomes. Preoperative data consisted of diagnosis (afterward confirmed by a histopathologic evaluation), sex, age, BMI, ASA status, and preoperative portal vein embolization. Perioperative variables were procedure date, open or minimally invasive surgery, type, and extension of liver resection (major resection when 3 or more contiguous segments were resected), one or two stages hepatectomy, use of Pringle maneuver, necessity and volume of transfusion, surgery time, need for ICU, length of hospital stay, postoperative morbidity according to the Dindo-Clavien classification, 90-day mortality.

Patients were followed according to the institutional protocol for each diagnosis. Overall Survival (OS) was defined as the time interval between the date of liver resection and the date of death or more recent contact during follow-up.

Survival was assessed using the Kaplan-Meier method and a comparison between the curves was performed with the log-rank test. Qualitative variables are presented as frequencies and percentages. Univariate associations between clinicopathologic qualitative variables and eras were examined using the χ2 test and/or Fisher's exact test. Quantitative variables are shown in mean values, median values, standard deviations, and ranges (minimum and maximum values). Data normality was evaluated using the Kolmogorov-Smirnov non-parametric test for quantitative variables. Comparison between the distribution of the quantitative variables between treatment groups was then completed using the Student's *t*-test (data with normal distribution) or Mann-Whitney test (data without normal distribution). A p-value of < 0.05 was considered statistically significant. Statistical analyses were performed with SPSS for Windows®, version 26.0 (IBM, Armonk, NY, USA).

## Results

**Fig. 1:**
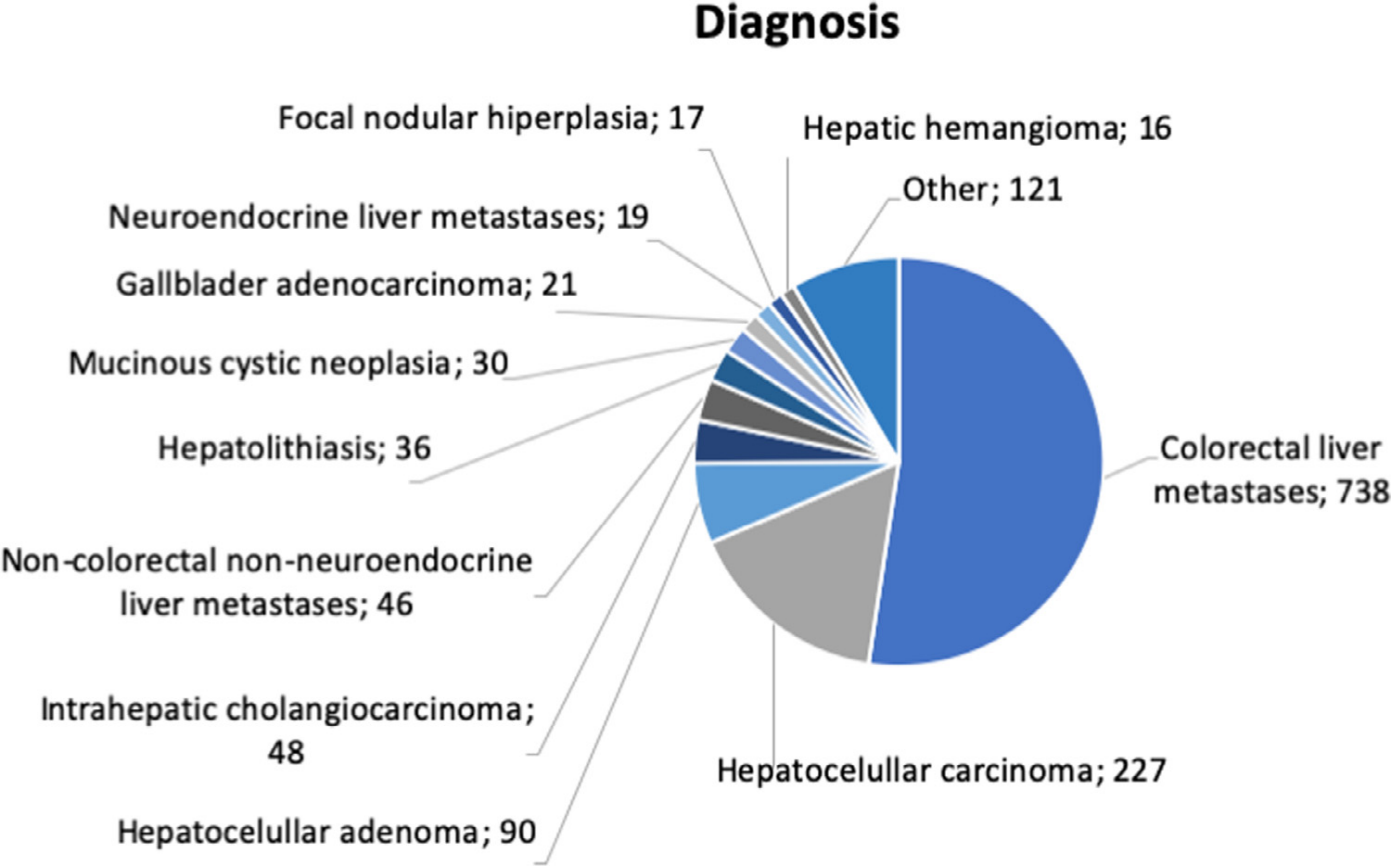
Preoperative diagnosis for patients submitted to liver resection (2000‒2020).

During the study period, 1409 liver resections were performed. Indications for liver resection are shown in Fig. 1. More than half (52.4%) of all liver resections were for CRLM, 16.1% for HCC, 6.4% for liver cell adenoma, and 3.4% for intrahepatic cholangiocarcinoma, 3.3% for non-colorectal non-neuroendocrine liver metastasis, 2.6% for intrahepatic lithiasis, 2.1% for mucinous cystic neoplasia. Table 1 summarizes preoperative and postoperative patients' characteristics.

**Table 1:** Preoperative and postoperative study population characteristics for patients submitted to liver resection (2000‒2020).

Variable	Characteristic		n (%)
Sex	Female		737 (52.3)
	Male		672 (47.7)
Age	< 70 years		1159 (82.3)
	≥ 70 years		250 (17.7)
BMI (Kg/m^2^)	Mean (SD)	26.2 (4.6)	
	Median (min‒max)	25.6 (15.0‒45.7)	
ASA status	I		293 (20.8)
	II		984 (69.8)
	III		129 (9.2)
	IV		3 (0.2)
Preoperative portal vein embolization	No		1372 (97.4)
	Yes		37 (2.6)
Era	Era 1		333 (23.6)
	Era 2		1076 (76.4)
Diagnosis	Colorectal liver metastases		738 (52.4)
	Hepatocellular carcinoma		227 (16.1)
	Other		444 (31.5)
Surgical approach	Open		986 (70.0)
	Minimally invasive resection		423 (30.0)
Type of resection	Right hepatectomy		277 (19.7)
	Left hepatectomy		143 (10.1)
	Bisegmentectomy 2‒3		154 (10.9)
	Bisegmentectomy 6‒7		66 (4.7)
	Right extended		30 (2.1)
	Left extended		4 (0.3)
	Other anatomical resections		187 (13.3)
	Wedge resections		548 (38.9)
Extension of resection	Major		473 (33.6)
	Minor		936 (66.4)
Two-stage hepatectomy	No		1372 (97.4)
	Yes		37 (2.6)
Pringle maneuver a	No		878 (64.0)
	Half-Pringle		198 (14.4)
	Pringle		296 (21.6)
Blood transfusion	No		1156 (82.0)
	Yes		253 (18)
Need for postoperative ICU	No		244 (17.3)
	Yes		1165 (82.7)
Length of hospital stay	Mean (SD)	8.9 (9.3)	
	Median (min‒max)	6 (0‒99)	
Perioperative morbidity (Dindo-Clavien)	0‒II		1202 (85.4)
	III‒IV		148 (10.5)
	V		59 (4.2)

SD, Standard Deviation; BMI, Body Mass Index; ASA, American Society of Anesthesiologists classification; Era 1 (2000‒2010); Era 2 (2011‒2020); ICU, Intensive Care Unit.

a 37 missing patients.

**Fig. 2:**
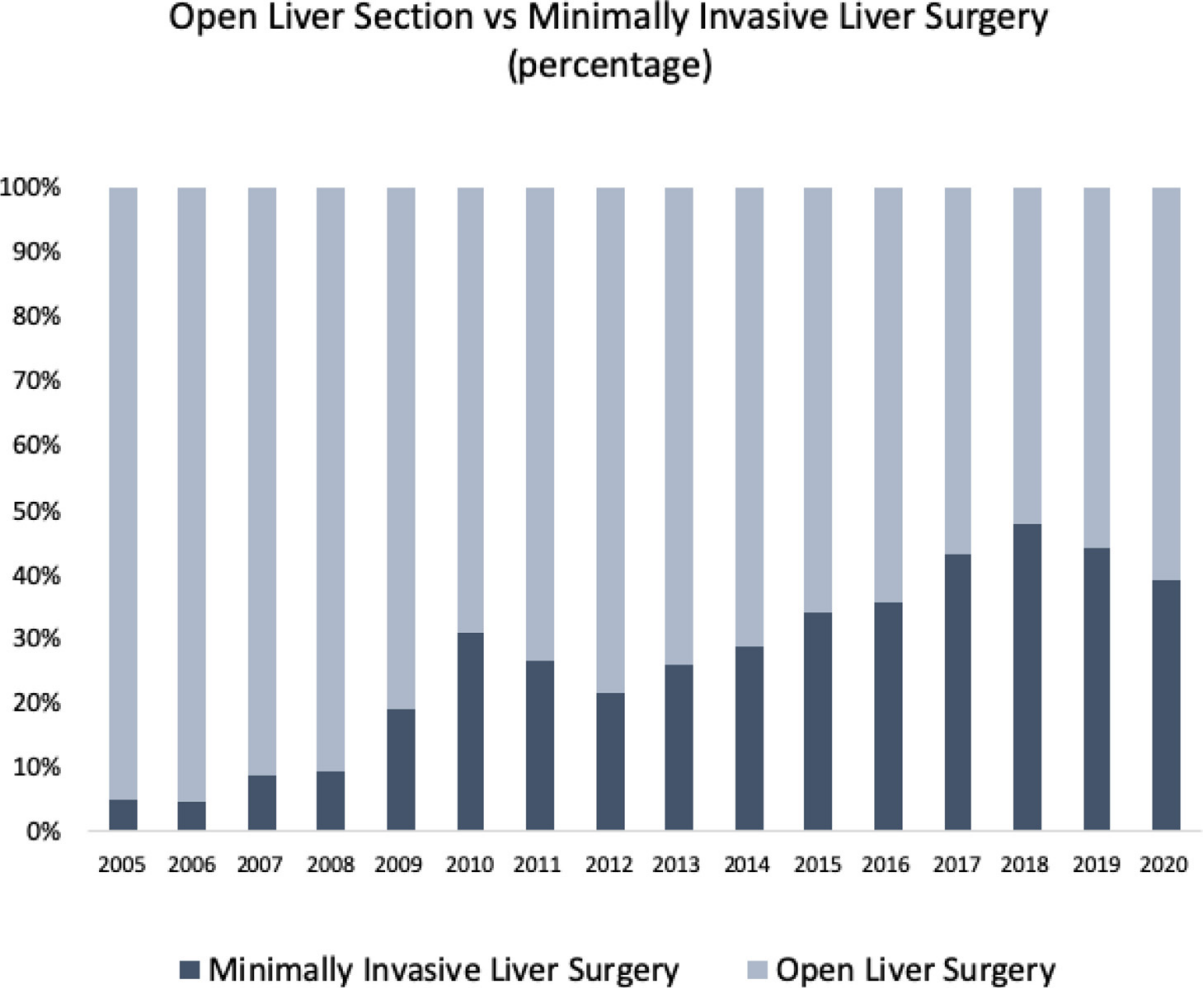
Percentage of open and minimally invasive liver resections.

When patients from the two Eras were compared, the authors observed on Era 2 a higher BMI, and significantly more: minimally invasive surgeries (Fig. 2), preoperative portal vein embolizations, Pringle maneuvers, and minor liver resections. On the other hand, less transfusion, less ICU necessity, and a shorter length of hospital stay were observed. Postoperative complications were considered severe (Clavien ≥III) in 207 patients (14.7%), and 90-day mortality was 4.2%. Morbidity and mortality rates between eras were not different. Table 2 summarizes the comparison between eras.

**Table 2:** Liver resections (n = 1409): comparison between eras.

			Era 1	Era 2	
			n = 333	n = 1076	
Variable		Total	n (%)	n (%)	p-value
Sex	Female	737	184 (55.3)	553 (51.4)	0.218
	Male	672	149 (44.7)	523 (48.6)	
Age	< 70 years	1159	274 (82.3)	885 (82.2)	0.989
	≥ 70 years	250	59 (17.7)	191 (17.8)	
BMI (Kg/m^2^)	Mean (min‒max)		25.6 (15.6‒41.2)	26.4 (15.0‒45.7)	**0.013**
	Median (SD)		25.1 (4.67)	25.9 (4.65)	
ASA status	I	293	72 (21.6)	221 (20.5)	0.093
	II	984	237 (71.2)	747 (69.4)	
	III	129	22 (6.6)	107 (9.9)	
	IV	3	2 (0.6)	1 (0.1)	
Preoperative portal vein embolization	No	1372	330 (99.1)	1042 (96.8)	**0.029**
	Yes	37	3 (0.9)	34 (3.2)	
Diagnosis	Colorectal metastases	738	167 (50.2)	571 (53.1)	0.095
	Hepatocellular carcinoma	227	67 (20.1)	160 (14.9)	
	Other	444	99 (29.7)	345 (32.0)	
Surgical approach	Open	986	290 (87.1)	696 (64.7)	**<0.001**
	Minimally invasive	423	43 (12.9)	380 (35.3)	
Type of resection	Right hepatectomy	277	95 (28.5)	182 (16.9)	**<0.001**
	Left hepatectomy	143	36 (10.8)	107 (9.9)	
	Bisegmentectomy 2‒3	154	36 (10.8)	118 (11.0)	
	Bisegmentectomy 6‒7	66	14 (4.2)	52 (4.8)	
	Other anatomical resections	221	68 (20.5)	153 (10.9)	
	Wedge resection	548	84 (25.2)	464 (43.1)	
Extension of resection	Major	473	149 (44.7)	324 (30.1)	**<0.001**
	Minor	936	184 (55.3)	752 (69.9)	
Two-stage hepatectomy	No	1372	322 (96.7)	1050 (97.6)	0.376
	Yes	37	11 (3.3)	26 (2.4)	
Use of Pringle maneuver^a^	No	878	230 (74.2)	648 (61.0)	**<0.001**
	Half-Pringle	198	36 (11.6)	162 (15.3)	
	Pringle	296	44 (14.2)	252 (23.7)	
Blood transfusion	No	1156	227 (68.2)	929 (86.3)	**<0.001**
	Yes	253	106 (31.8)	147 (13.7)	
Need for ICU	No	244	27 (8.1)	217 (20.2)	**<0.001**
	Yes	1165	306 (91.9)	859 (79.8)	
Length of hospital stay	Mean (min‒max)		9.7 (0-71)	8.7 (0-99)	**<0.001**
	Median (SD)		7 (8.77)	6 (9.39)	
Perioperative morbidity	Severe	207	57 (17.1)	150 (13.9)	0.152
	Minor	1202	276 (82.9)	926 (86.1)	
Perioperative mortality	No	1350	320 (96.1)	1030 (95.7)	0.768
	Yes	59	13 (3.9)	46 (4.3)	

SD, Standard Deviation; BMI, Body Mass Index; ASA, American Society of Anesthesiologists classification; Era 1 (2000‒2010); Era 2 (2011‒2020); ICU, Intensive Care Unit.

a 37 missing patients.

**Table 3:** Comparison between eras (colorectal liver metastases).

		Era 1	Era 2	p-value
		n = 167	n = 571	
Variable		n (%)	n (%)	
Sex	Female	80 (47.9%)	269 (47.1%)	0.856
	Male	87 (52.1%)	302 (52.9%)	
Age	< 70 years	130 (77.8%)	475 (83.2%)	0.114
	≥ 70 years	37 (22.2%)	96 (16.8%)	
BMI (Kg/m^2^)	Mean (min‒max)	25.6 (16.9‒37.3)	26.3 (15‒41.8)	0.107
	Median (SD)	25 (4.25)	25.0 (4.47)	
ASA status	I	29 (17.4%)	95 (16.6%)	0.058
	II	133 (79.6%)	428 (75.0%)	
	III	5 (3.0%)	48 (8.4%)	
Largest tumor size	Mean (min‒max)	4.67 (0.40-23.5)	3.29 (0.2-16.1)	**<0.001**
	Median (SD)	3.90 (3.73)	2.75 (2.34)	
Number of nodules	1‒3	125 (80.1%)	378 (73.7%)	0.103
	> 3	31 (19.9%)	135 (26.3%)	
Bilateral nodules	No	115 (72.3)	326 (59.4)	**0.003**
	Yes	44 (27.7)	223 (40.6)	
Neoadjuvant chemotherapy	No	82 (51.6)	125 (22.8)	**<0.001**
	Yes	77 (48.4)	424 (77.2)	
Preoperative portal vein embolization	No	165 (98.8%)	549 (96.1%)	0.133
	Yes	2 (1.2%)	22 (3.9%)	
Surgical approach	Open	157 (94.0%)	429 (75.1%)	**<0.001**
	Minimally invasive	10 (6.0%)	142 (24.9%)	
Type of resection	Right hepatectomy	56 (33.5%)	96 (16.8%)	**<0.001**
	Left hepatectomy	19 (11.4%)	41 (7.2%)	
	Bisegmentectomy 2‒3	14 (08.4%)	43 (07.5%)	
	Bisegmentectomy 6‒7	8 (04.8%)	29 (05.1%)	
	Other anatomical resections	34 (20.3%)	83 (14.5%)	
	Wedge resection	36 (21.6%)	279 (48.9%)	
Extension of resection	Major	85 (50.9%)	157 (27.5%)	**<0.001**
	Minor	82 (49.1%)	414 (72.5%)	
Two-stage hepatectomy	No	157 (94.0%)	546 (95.6%)	0.408
	Yes	10 (06.0%)	25 (04.4%)	
Use of Pringle maneuver^a^	No	111 (73.0%)	348 (62.1%)	**0.035**
	Half-Pringle	12 (07.9%)	76 (13.6%)	
	Pringle	29 (19.1%)	136 (24.3%)	
Blood transfusion	No	109 (65.3%)	504 (88.3%)	**<0.001**
	Yes	58 (34.7%)	67 (11.7%)	
Need for ICU	No	12 (07.2%)	126 (22.1%)	**<0.001**
	Yes	155 (92.8%)	445 (77.9%)	
Length of hospital stay	Mean (min‒max)	9.4 (2-71)	8.9 (1-99)	**0.019**
	Median (SD)	7 (8.90)	6 (9.40)	
Perioperative morbidity	Severe	23 (13.8%)	74 (13.0%)	**0.784**
	Minor	144 (86.2%)	497 (87.0%)	
Perioperative mortality	No	161 (96.4%)	547 (95.8%)	**0.725**
	Yes	6 (03.6%)	24 (04.2%)	

SD, Standard Deviation; BMI, Body Mass Index; ASA, American Society of Anesthesiologists classification; Era 1 (2000‒2010); Era 2 (2011‒2020); ICU, Intensive Care Unit.

a 26 missing patients.

### Colorectal liver metastases

Seven hundred thirty-eight liver resections for Colorectal Liver Metastasis (CRLM) in 708 patients were performed. In Era 2 there were significantly more patients with bilateral metastases, but with smaller sizes. Regarding the number of metastatic nodules, the authors observed more multinodular cases in Era 2 (26.3% vs. 19.9%) however the difference was not significant (p = 0.103). Moreover, patients received more neoadjuvant chemotherapy in Era 2.

A comparison between eras was made showing in Era 2 significantly less transfusion, the necessity of ICU, and a shorter length of hospital stay. From a technical point of view, in Era 2 the authors observed more: pedicle clamping maneuvers, minimally invasive surgeries (6% vs. 24.9%; p < 0.001); and minor (49.1% vs. 72.5%; p < 0.001) or wedge resections (21.6% vs. 48.9%; p < 0,001) were employed.

**Fig. 3:**
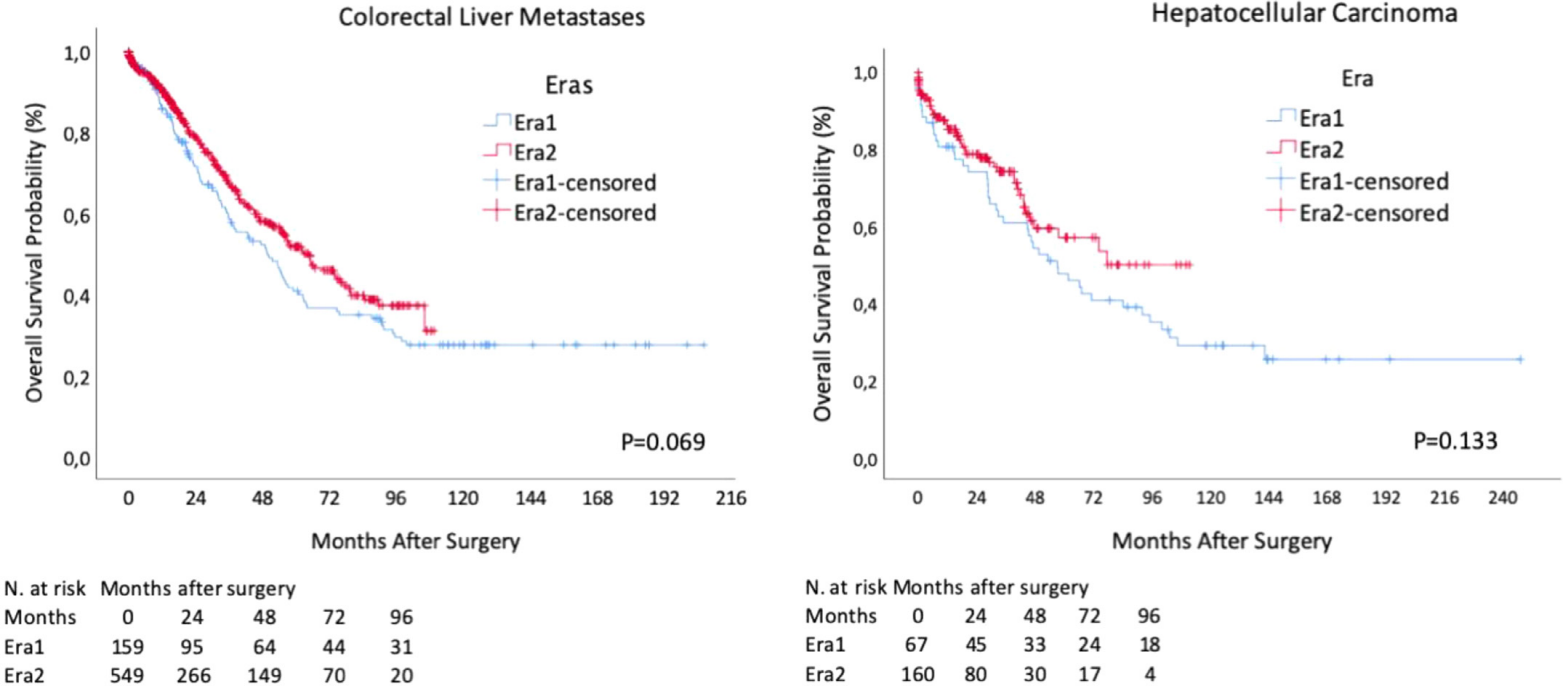
(A) CRLM resection overall survival according to different eras; (B) HCC resection overall survival according to different eras.

The whole CRLM group OS was 89.4%, 65.3%, and 48.2% at 1, 3 , and 5 years, respectively. When comparing both eras, OS at 1, 3 , and 5 years in Era 1 was 86.3%, 58.4%, and 40.7%, respectively; and in Era 2 OS was 90.4%, 67.9%, and 51.5% at 1, 3, and 5 years, respectively. OS was not different between eras (p = 0.069) (Fig. 3A).

### Hepatocellular carcinoma

Two hundred twenty-seven resections for Hepatocellular Carcinoma (HCC) were performed. In the HCC group, a comparison between eras was made showing in Era 2 significantly more minimally invasive surgeries and fewer transfusions, less necessity of ICU, and a shorter length of hospital stay (Table 4).

**Table 4:** Comparison between eras (hepatocellular carcinoma).

		Era 1	Era 2	p-value
		n = 67	n = 160	
Variable		n (%)	n (%)	
Sex	Female	27 (40.3%)	43 (26.9%)	**0.046**
	Male	40 (59.7%)	117 (73.1%)	
Age	<70 years	51 (76.1%)	115 (71.9%)	0.511
	≥70 years	16 (23.9%)	45 (28.1%)	
BMI (Kg/m^2^)	Mean (min‒max)	25.6 (15.6‒38.2)	25.5 (16.4‒36.7)	0.934
	Median (SD)	25.5 (4.94)	25.1 (4.22)	
ASA status	I	5 (07.5%)	13 (08.1%)	0.930
	II	49 (73.1%)	116 (72.5%)	
	III	12 (17.9%)	30 (18.8%)	
	IV	1 (01.5%)	1 (00.6%)	
Largest tumor size	Mean (min‒max)	6.8 (0.5‒24.0)	5.9 (0.5‒26.0)	0.145
	Median (SD)	5.0 (5.06)	4.0 (4.82)	
Number of nodules	1	54 (80.6%)	140 (87.5%)	0.17
	> 1	13 (19.4%)	20 (12.5%)	
Preoperative portal vein embolization	No	66 (98.5%)	155 (96.9%)	0.484
	Yes	1 (01.5%)	5 (03.1%)	
Surgical approach	Open	53 (79.1%)	59 (36.9%)	**<0.001**
	Minimally invasive	14 (20.9%)	101 (63.1%)	
Type of resection	Right hepatectomy	16 (23.9%)	28 (17.5%)	0.913
	Left hepatectomy	4 (06.0%)	12 (07.5%)	
	Bisegmentectomy 2‒3	9 (13.4%)	26 (16.3%)	
	Bisegmentectomy 6‒7	3 (4.5%)	12 (07.5%)	
	Other anatomical resections	13 (19.4%)	28 (17.4%)	
	Wedge resection	22 (32.8%)	54 (33.8%)	
Extension of resection	Major	23 (34.3%)	46 (28.8%)	0.405
	Minor	44 (65.7%)	114 (71.3%)	
Use of Pringle maneuver^a^	No	43 (66.2%)	89 (56.0%)	0.292
	Half-Pringle	13 (20.0%)	35 (22.0%)	
	Pringle	9 (13.8%)	35 (22.0%)	
Blood transfusion	No	46 (68.7%)	138 (86.3%)	**0.002**
	Yes	21 (31.3%)	22 (13.8%)	
Need for ICU	No	1 (1.5%)	15 (09.4%)	**0.034**
	Yes	66 (98.5%)	145 (90.6%)	
Length of hospital stay	Mean (min‒max)	12.9 (0‒54)	8.7 (1‒49)	**<0.001**
	Median (SD)	8 (11.31)	5 (8.49)	
Perioperative morbidity	Severe	16 (23.9%)	24 (15.0%)	0.109
	Minor	51 (76.1%)	136 (85.0%)	
Perioperative mortality	No	61 (91.0%)	149 (93.1%)	0.587
	Yes	6 (9.0%)	11 (06.9%)	

SD, Standard Deviation; BMI, Body Mass Index; ASA, American Society of Anesthesiologists classification; Era 1 (2000‒2010); Era 2 (2011‒2020); ICU, Intensive Care Unit.

a 3 missing patients.

OS was 83.9%, 68.7%, and 52.8% at 1, 3 and 5 years, respectively. When comparing both eras, OS at 1, 3 and 5 years in Era 1 was 80.3%, 60.6%, and 47.4%, respectively; and in Era 2 OS was 85.5%, 73.8%, and 56.8% at 1, 3 and 5 years, respectively. OS was not different between eras (p = 0.133) (Fig. 3B).

## Discussion

In the last two decades, liver resection has evolved from a high mortality complex procedure to a routine standardized operation increasingly employed. Liver resection is now established as the most effective treatment for selected patients with primary and secondary hepatic malignancy and benign diseases.^2^^,^^4^ This evolution is a result of the spreading of dedicated liver surgery units in a multidisciplinary environment with improvements in perioperative care leading to lower morbidity and mortality rates. The mortality decrease associated with the significant decrease in blood transfusion in oncologic liver surgery represents an advance in surgical care and impacts the indications for liver resection.^25^^,^^26^

In the last 20 years, the present group has performed 1409 liver resections experiencing the evolution of liver surgery. The main indications for liver resection were CRLM, HCC, liver cell adenoma, and intrahepatic cholangiocarcinoma. The authors have looked at the entire cohort of patients to evaluate the changes in the last two decades, and therefore as a subgroup analysis, we evaluated the most frequent indications for liver resection, CRLM, and HCC.

Looking at the present data, patients in Era 2 presented a higher BMI, reflecting a world tendency. In fact, since 1980 the prevalence of obesity has increased twofold in more than 70 countries and has risen in most other countries.^27^

In Era 2, more parenchyma-sparing resections were employed. These techniques are an important advance in oncologic liver surgery because they improve the safety of the procedure by decreasing the risk of postoperative liver failure.^28^ Moreover, in patients with CRLM, it allows a novel resection in cases of recurrence. In this context, the authors have performed significantly fewer major liver resections, especially fewer right hepatectomies in Era 2.

After the beginning of the quality program in liver surgery, the authors noticed that patients with CRLM submitted to neoadjuvant oxaliplatin-based chemotherapy needed more blood transfusions. This fact is probably due to the sinusoidal congestion (“blue liver”) caused by oxaliplatin,^29^ leading to more bleeding during liver transection. Consequently, in the last years (Era 2), the authors have employed more intermittent pedicle clamping (Pringle maneuver) during parenchyma transaction resulting in lower transfusion rates. For non-anatomical resections, especially on the right lobe of the liver, the authors employed a selective pedicle clamping (half-Pringle) as reported elsewhere.^30^ From an oncological point of view, the avoidance of blood transfusion impacts positively because many studies showed a negative impact on survival for patients who received a transfusion.^25^^,^^31^

Laparoscopic liver resections have reached increasing acceptance for the treatment of benign and malignant liver lesions over the last two decades.^32^^,^^33^ It offers better perioperative outcomes with less intraoperative bleeding and lower rates of postoperative complications without compromising oncologic results. Moreover, due to the low invasiveness, results in better recovery and shortening of hospital stay.^32^ In the present series, the rate of minimally invasive surgeries in the last era presented a threefold increase (12.9% to 35.3%). Most specialized hepatobiliary centers adopted the minimally invasive approach as reported in recent South American and European surveys where the proportion between minimally invasive and open liver resection ranged from 10% to 29%.^34^^,^^35^

In this series, a decrease in ICU needs and a shorter hospital stay are a result of multiple factors such as better patient selection and perioperative care, a parenchyma sparing approach, and the increasing use of minimally invasive surgery. These factors, associated with a lower bleeding rate observed in the last decade, can also lead to a cost reduction. The mortality rate (4.2%) observed in the present study did not change between eras and is in accordance with other large series worldwide.^2^^,^^6^

In this study, the authors focused on CRLM and HCC, the main indications for liver resection in our experience and worldwide.

All CRLM cases were discussed in a multidisciplinary meeting, and almost all patients were subjected to perioperative oxaliplatin-based systemic chemotherapy. In Era 1 chemotherapy was preferably delivered after liver resection. Neoadjuvant chemotherapy is mostly employed in patients with unfavorable prognostic factors to eliminate micrometastatic disease and understand tumor biology by evaluating response rates.^36^^,^^37^ In Era 2 when more patients with multiple (not significantly different) and bilateral diseases were treated, 77.2% were submitted to preoperative chemotherapy, significantly more than in Era 1 (48.4%). As a result of a better follow-up and surveillance for patients with colorectal cancer, and the use of preoperative systemic treatment, the authors observed in Era 2 patients with smaller tumor sizes.

In the last years (Era 2), the authors adopted the concept of parenchyma sparing resection for CRLM with significantly more minor and wedge resections. Mise et al.^38^ and Torzilli^39^ have shown that preserving liver parenchyma does not increase local recurrence. Moreover, an increase in survival was observed in patients submitted to parenchyma sparing resection due to the possibility of performing new treatments in case of recurrence (re-hepatectomy or ablation).

There was a significant increase in minimally invasive procedures when comparing Eras. For CRLM this increase was from 6% to 24.9% in Era 1 and Era 2, respectively. Indeed, the Oslo group has reported the first prospective randomized trial comparing open and laparoscopic resection of CRLM and showed less postoperative complications and shorter hospital stay in the laparoscopic group.^40^

The 5-year survival following CRLM resection was 40.7% in Era 1 and 51.5% in Era 2. This increase, despite not being significant, reflects a better staging, the evolution of chemotherapy regimens, and the use of modern surgical strategies (parenchyma sparing, staged liver resections, portal vein embolization, ALPPS). It should be noted that in Era 2 the authors operated on patients with more advanced disease (more nodules and bilateral disease), and despite this, the results improved, showing an advance in the selection and treatment strategies for CRLM.

Despite the debate between resection versus liver transplantation, in the present context of a lack of donors and a long waiting list time, HCC resection became an excellent curative option, especially in patients with preserved liver function. Moreover, resection can provide treatment for patients not candidates for transplant. All cases were discussed in a multidisciplinary meeting with hepatologists, oncologists, transplant surgeons, radiologists, and liver surgeons to define the best treatment strategy.

From all indications of laparoscopic liver resection, patients with HCC are those who benefit most from the minimally invasive approach.^41^, ^42^, ^43^ In addition to the benefits already mentioned, a lower incidence of postoperative ascites was consistently observed following the minimally invasive resection in patients with chronic liver disease.^19^, ^20^, ^21^, ^22^, ^23^, ^24^, ^25^, ^26^, ^27^, ^28^, ^29^, ^30^, ^31^, ^32^, ^33^, ^34^, ^35^, ^36^, ^37^, ^38^, ^39^, ^40^, ^41^, ^42^, ^43^, ^44^ This is probably a consequence of the preservation of the abdominal wall and umbilical ligament collateral venous circulation. Moreover, in cases of recurrence, salvage transplantation can be more easily performed following laparoscopic liver resection when compared to open surgery due to fewer adhesions.^45^

There was also a significant increase in minimally invasive procedures for HCC, from 20.9% to 63% in Era 1 and Era 2, respectively. The increase in the minimally invasive approach resulted in fewer transfusions, a lower necessity of ICU, and shorter hospital stay.

The 5-year survival following HCC resection was 47.4% in Era 1 and 56.8% in Era 2. Despite not being significant, the improvement in survival rates was probably due to a better staging (modern imaging techniques), and a rigorous selection of patients.

The present study's results are in accordance with the most important specialized hepatobiliary groups in the world.^41^^,^^43^^,^^46^ The multidisciplinary approach has provided much better results than those observed in the past, allowing an expansion of the limits both in the indication and in liver surgery itself. In the last decade, significantly more minimally invasive surgeries were done, and less bleeding and better perioperative results were observed.

Surgery remains the cornerstone for the curative treatment of primary and metastatic liver tumors but, to achieve excellent results, it is recommended that this complex procedure should be performed in a multidisciplinary environment.

## Authors' contributions

Paulo Herman: Conceived the idea and wrote the paper.

Gilton Marques Fonseca: Collected data, performed the statistical analysis, and helped in the paper writing.

Fabricio Ferreira Coelho: Reviewed and made suggestions during the paper writing.

Jaime Arthur Pirolla Kruger: Collected data and reviewed paper writing.

Fabio Ferrari Makdissi: Reviewed and made suggestions during the paper writing.

Vagner Birk Jeismann: Reviewed and made suggestions during the paper writing.

Flair José Carrilho: Reviewed the paper.

Luiz Augusto Carneiro D´Albuquerque: Reviewed the paper.

Sergio Carlos Nahas: Reviewed the paper.

## Conflicts of interest

The authors declare no conflicts of interest.
